# A comparative analysis of secreted protein disulfide isomerases from the tropical co-endemic parasites *Schistosoma mansoni* and *Leishmania major*

**DOI:** 10.1038/s41598-019-45709-8

**Published:** 2019-07-02

**Authors:** Adriana E. Miele, Sofiane Badaoui, Lorenzo Maugliani, Romain Salza, Giovanna Boumis, Silvia Chichiarelli, Bertrand Duclos, Sylvie Ricard-Blum

**Affiliations:** 1 Department Biochemical Sciences, Sapienza University of Rome, P.le Aldo Moro 5, Rome, 00185 Italy; 2 ICBMS UMR 5246, CNRS - Université de Lyon, 43 Boulevard du 11 Novembre 1918, Villeurbanne, cedex 69622 France

**Keywords:** Chaperones, Parasitic infection, SAXS

## Abstract

The human parasites *Schistosoma mansoni* and *Leishmania major* are co-endemic and a major threat to human health. Though displaying different tissue tropisms, they excrete/secrete similar subsets of intracellular proteins that, interacting with the host extracellular matrix (ECM), help the parasites invading the host. We selected one of the most abundant proteins found in the secretomes of both parasites, protein disulfide isomerase (PDI), and performed a comparative screening with surface plasmon resonance imaging (SPRi), looking for ECM binding partners. Both PDIs bind heparan sulfate; none of them binds collagens; each of them binds further ECM components, possibly linked to the different tropisms. We investigated by small-angle X-ray scattering both PDIs structures and those of a few complexes with host partners, in order to better understand the differences within this conserved family fold. Furthermore, we highlighted a previously undisclosed moonlighting behaviour of both PDIs, namely a concentration-dependent switch of function from thiol-oxidoreductase to holdase. Finally, we have tried to exploit the differences to look for possible compounds able to interfere with the redox activity of both PDI.

## Introduction

Vector borne diseases (including malaria, Dengue, human trypanosomiasis, leishmaniasis, schistosomiasis and Chagas disease) are major threats to human health worldwide^1^. Despite the diversity of pathogens, which range from virus to protists to worms, all of them do not induce stable immunity, therefore vaccines based on humoral response have a low protection rate, when available^2–5^. The life cycles of these parasites were defined over 100 years ago, however the strategies they use to optimize their successful transmission are only starting to be understood at the molecular level. Parasites are now known to monitor their environment in both their host and vector and in response to other parasites. This allows them to adapt their developmental cycles and to counteract any unfavourable conditions they encounter^6^.

Understanding the molecular and immunological mechanisms of the crosstalk between the parasite and the host is a prerequisite for rational vaccine discovery and drug development.

Here we focus on two human co-endemic parasites, *Leishmania major* and *Schistosoma mansoni*, because before reaching their ultimate target they both contact the extracellular matrix (ECM) of the host, especially in the dermis^7,8^. *L*. *major*, one of the causative agents of cutaneous leishmaniasis, is an intracellular eukaryotic parasite targeting macrophages. *S*. *mansoni*, the widest spread agent of intestinal schistosomiasis, is a multicellular digenean extracellular trematode, capable to finely tune the inflammatory response mediated by lymphocytes and macrophages.

We have focussed our research on protein disulfide isomerases (PDI), which are moonlighting proteins, abundantly found in both parasites secretomes and are putative therapeutic/vaccine targets^9–15^. PDIs are widespread and highly conserved proteins, belonging to the large thioredoxin (Trx) superfamily, and are modular proteins containing between 2 and 4 Trx-like domains^16,17^. They catalyse the oxidation and/or shuffling of disulphides in substrate proteins and are involved in oxidative folding, mediated by their disulphide bond of high reduction potential and a thiol group of low pKa. The active site is composed of a CXXC motif and by a cleft capable to host a variety of large peptidyl substrates^16,17^. Most of the members of the PDI family also contain the endoplasmic reticulum (ER) retention signature (K/HDEL) and a non-canonical signal peptide for export. PDIs from pathogens have recently come centre stage for their “*Janus*” behaviour^18^. Indeed these proteins can exist in two forms: a cytosolic/ER one and a tegument/extracellular one, independently of the presence of a signal peptide for secretion. They act intracellularly as oxidative chaperones, but when secreted by the parasites they interact with host proteins, possibly modulating the host response. Sometimes they are also helped by the host PDI as documented for *L*. *chagasi* and Dengue virus^19,20^.

The human extracellular matrix (ECM) is composed of proteins, proteoglycans and glycosaminoglycans (GAGs), and plays structural and functional roles also in host-parasite interactions^7,21–25^. ECM undergoes remodelling and dynamic reorganization in both physiological and pathological conditions, including parasitic diseases^23,26–28^, which trigger the release of bioactive fragments of ECM proteins, called matricryptins^29^. These last regulate diverse processes, including angiogenesis, tumor growth and growth-factor mediated signalling pathways^29,30^. Moreover, the matrycryptin endostatin binds intact *Leishmania* promastigotes, thus contributing to their interactions with the host ECM^23^.

In this study we present the structure, enzymatic activity and interactions established by two parasite PDIs, namely *L*. *major* PDI (LmPDI) and *S*. *mansoni* PDI (SmERp60), with the ECM of the human host. To the best of our knowledge this is the first time these two enzymes are comparatively characterized. The structure of both proteins was determined by SAXS and their interactions with host proteins and GAG arrays were identified by surface plasmon resonance imaging (SPRi), a method we have successfully used to build the interaction repertoire between 24 strains of *Leishmania* and the human host ECM^23^. We show that both proteins are monomeric, as the two major PDIs from the human host (HsPDIA1 and HsPDIA3/HsERp57). Both LmPDI and SmERp60 are able to reduce insulin disulphide bonds and di-eosin glutathione disulphide (di-eosin-GSSG), and both display a previously undisclosed concentration-dependent switch of function from oxido-reductase to holdase. Interestingly, LmPDI has a temperature-dependent redox activity and undergoes a reversible cold denaturation, similarly to what reported for *Saccharomyces cerevisiae* PDI (ScPDI), the only other eukaryotic full length PDI structure present in the PDB^31,32^. Finally, the structure in solution of the complexes formed with interacting GAGs was also investigated by size exclusion chromatography (SEC) coupled to SAXS (SEC-SAXS).

## Results

### Structural features of LmPDI and SmERp60

The genes encoding LmPDI and SmERp60 were cloned, the corresponding proteins were expressed in *E*. *coli* and purified to homogeneity by affinity chromatography, as reported in the Methods section. Both proteins were concentrated by ultrafiltration and then either used straight away or stored at 4 °C.

**Figure 1:**
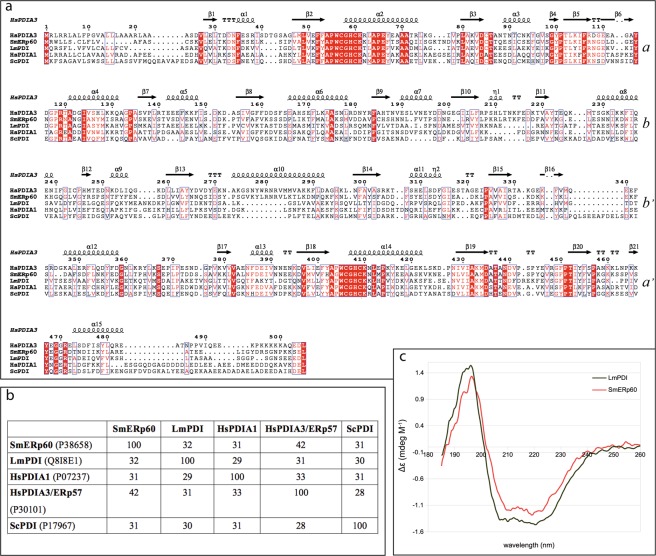
Primary and secondary structure comparison of *S*. *mansoni* and *L*. *major* Protein Disulfide Isomerases. Panel (**a**) Primary structure alignment of LmPDI and SmERp60 with the human host closest homologues (HsPDIA3/ERp57, HsPDIA1) and with *Saccharomyces cerevisiae* PDI (ScPDI). The alignment was performed with ClustalOmega on the EBI server^76^ and rendered with ESPript 3.x^77^, the secondary structure of HsPDIA3/ERp57, based on the crystallographic structure (3F8U^33^), is displayed on top of the alignment. Each line roughly represents one Trx-like domain, indicated as *a*, *b*, *b*′ and *a*′ (see text). Panel (**b**) Pairwise sequence identity matrix of human (Hs), parasites (Lm and Sm) and yeast (Sc) PDIs as computed by ClustalOmega on the EBI server^76^, values are % of identical residues. The codes within the parenthesis are the UniProtKB accession numbers of each protein^78^. HsPDIA1, *Homo sapiens* PDI isoform A1; HsPDIA3/ERp57, *H*. *sapiens* PDI isoform A3, also known as ERp57; LmPDI, *Leishmania major* PDI; SmERp60, *Schistosoma mansoni* Endoplasmic Reticulum protein 60; ScPDI, *Saccharomyces cerevisiae* PDI. Panel (**c**) Secondary structure analysis of the recombinant proteins by circular dichroism. Spectra of SmERp60 (red) and LmPDI (green) at 25 °C, after buffer subtraction and averaging of seven sequential acquisitions on a Jasco J-810 instrument, are presented.

The sequences of the Trx-like domains are well conserved among LmPDI, SmERp60 and the human host most abundant PDIs (HsPDIA3/ERp57, UniProtKB P30101; HsPDIA1, UniprotKB P07237) (Fig. 1a,b), but the loops connecting the four Trx-like domains are less conserved. In the alignment shown in Fig. 1a we have also included the baker’s yeast PDI (ScPDI), which is the only other eukaryotic full length PDI present in the PDB (2B5E and 3BOA) and the only one reported to be temperature sensitive^31,32^. In all PDIs the two external domains (conventionally named *a* and *a*′^16,17^) contain the catalytic Cys pair and display a higher sequence identity (Fig. 1a), while the two central ones (*b* and *b*′) are catalytically inactive and more variable in the sequence, leading to an overall low sequence identity among the 4 proteins (Fig. 1b). It is worth noticing that the linker between *b* and *b*′ and the one between *b*′ and *a*′ display fewer identical residues and different lengths (Fig. 1a, sequence between β12 and β13 and between β15 and α12). The secondary structure of recombinant LmPDI and SmERp60 was analysed by circular dichroism (CD, Fig. 1c). The content of beta strands and coiled portions computed from the CD spectra was in line with that inferred from HsPDIA3/ERp57 crystal structure (PDB 3F8U^33^); however, there was a higher content of alpha helix in LmPDI with respect to SmErp60 and HsPDIA3/ERp57 (Table 1), making it more similar to the secondary structure content of the yeast homologue ScPDI (Table 1) computed from the crystal structure (PDB 2B5E^31^).

**Table 1:** Secondary structure content (%) calculated from deconvolved CD spectra performed on the DichroWeb server^65^, using CDSST3 and SELCON3 with sp175 set^66^.

	SmERp60	LmPDI	HsPDIA3/ERp57	ScPDI
α helix	28	41	*32*	*38*
β-strand	26	18	*21*	*19*
β-turn	17	12	*15*	*12*
unstructured (including random coil)	28	26	*32*	*30*

For comparison, secondary structures of host HsPDIA3/ERp57 and yeast ScPDI derived from the respective crystal structures (PDB 3F8U^33^ and 2B5E^31^) using DSSP on the 2Struc database^79^ are also reported in italics.

**Figure 2:**
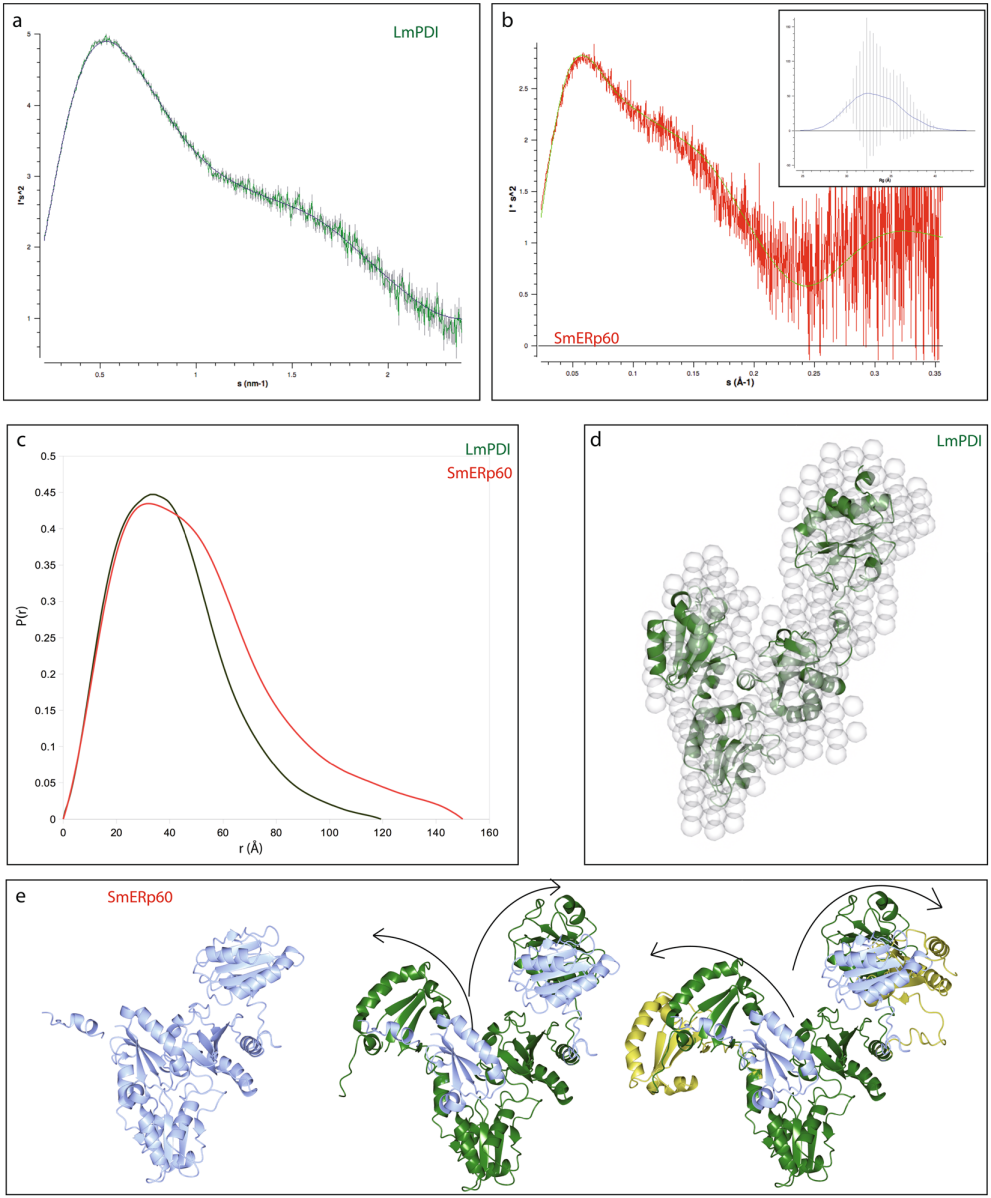
SAXS analysis of LmPDI and SmERp60 size and shape. Panel (a) Kratky plot of LmPDI (green) scattering curve, fitted with GNOM on the ATSAS suite^69^. Panel (b) Kratky plot of SmERp60 (red) scattering curve, fitted with the combination of conformations found after the deconvolution analysis. In the insert is shown the distribution of Rg of the particles, as computed by EOM^34^. Panel (c) Superposition of the pair probability distribution functions of SmERp60 (red) and LmPDI (green), as computed with the ATSAS suite^69^. Panel (d) *Ab initio* SAXS envelope of LmPDI, calculated by DAMIN/DAMMIF^72,73^, with the yeast ScPDI structure (2B5E) docked with Supcomb, all programs were run from the ATSAS suite^69^. Panel (e) Superimposed models of the 3 possible conformations of SmERp60 (blue, green and gold) compatible with SAXS data fitting after EOM^34^. The crystal structures of HsERp57/PDIA3 (3F8U^33^) and HsPDIA1 (4EL1 and 4EKZ^36^) have been used as template models to fit the deconvolved SAXS spectra. 3D images were produced with CCP4MG^75^.

In order to get more information into the size and shape of LmPDI and SmERp60, we used SAXS both under flow in a sample changer (Fig. 2) and coupled to a HPLC size exclusion chromatography (SEC-SAXS; Supplementary Fig. S2). The SAXS data (summarised in Table 2) suggested that both proteins are monomers, with LmPDI being compact, and SmERp60 highly flexible. In fact scattering data from LmPDI were fitted with a compact elliptical particle (as shown in the Kratky plot representation in Fig. 2a), whose radius of gyration (Rg) is 3.8 nm and the maximal particle dimension (D_max_) is 12 nm. In contrast, the scattering data of SmERp60 were compatible with a multi-domain protein carrying flexible linkers (Fig. 2b). LmPDI is as compact as the protein from baker’s yeast (ScPDI) [PDB: 2B5E^31^], whose monomer can be fitted into the reconstructed LmPDI’s envelope (χ^2^ = 1.03, Fig. 2d). Interestingly, both LmPDI and ScPDI have a temperature sensitive structure: in fact the yeast protein has been crystallized as dimer at room temperature (22 °C) and as a monomer at 4 °C (PDB 3BOA and 2B5E, respectively^31,32^). LmPDI is a compact monomer at 20 °C, while unfolded and partially aggregated at 5 °C (Supplementary Fig. S1). Therefore it undergoes a cold denaturation, with loss of function that can be rescued by heating the sample at 37 °C (see next paragraph). This refolding step is complete when the purified protein is conserved at 4 °C, while the functional rescue is very low (if any) after freeze-thaw at −20 °C.

**Table 2:** Summary of SAXS analysis on the recombinant untagged parasite proteins LmPDI and SmERp60 collected at BM29^68^ with the automatic sample changer, under continuous flow.

	LmPDI	SmERp60
Concentration range (mg ml^−1^)	0.5–6	0.5–5
Temperature (K)	293	293
**Structural parameters**		
Rg (nm) [from P(r)]	3.5 ± 0.3	3.98 ± 0.1
Rg (nm) [from Guinier]	3.2 ± 0.2	3.88 ± 0.1
D_max_ (nm)	11.0 ± 1	13.91 ± 0.5
Porod volume estimate (nm^3^)	95 ± 15	92 ± 25
Dammif excluded volume (nm^3^)	140 ± 15	141 ± 18
**Molecular mass determination**		
Molecular mass (kDa) [from Porod invariant]^69^	56 ± 15	76 ± 15
Molecular mass (kDa) [from SAXSMoW website]^80^	58	77
Theoretical molecular mass (kDa) [from ProtParam]^81^	51.8	54.5

The buffer (50 mM Tris/HCl, 150 mM NaCl, pH 7.4) was recirculated before and after every sample. Exposure time 1 s, beam attenuation (to avoid radiation damage) 50%. Ten images per each concentration were collected and averaged. The Molecular mass was derived thanks to the calibration with a BSA stock solution.

The conformational flexibility of SmERp60 was explored by the Ensemble Optimization Method (EOM^34^), suggesting that it might adopt an ensemble of conformations (insert of Fig. 2b) with Rg ranging from 3 to 6 nm and D_max_ from 11 to 16 nm. We also used single value deconvolution to extract the 3 most representative conformations, which were interpreted by fitting the deposited crystal structures of HsPDIA3/ERp57 (2H8L,^35^, 3F8U^33^,) and HsPDIA1 (4EKZ, 4EL1^36^,), because they shared the highest sequence identity with SmERp60 (Fig. 1a,b). The results suggested a butterfly movement of the external *a* and *a*′ catalytic domains with respect to the central fixed *b-b*′ domains, from a close to an open conformation (Fig. 2e left to right**)**. The importance of such a flexibility of PDI superfamily to better adapt to diverse substrates was also recently reviewed by Freedman and co-workers^17^.

### Disulfide reductase and chaperone activity of LmPDI and SmERp60

**Figure 3:**
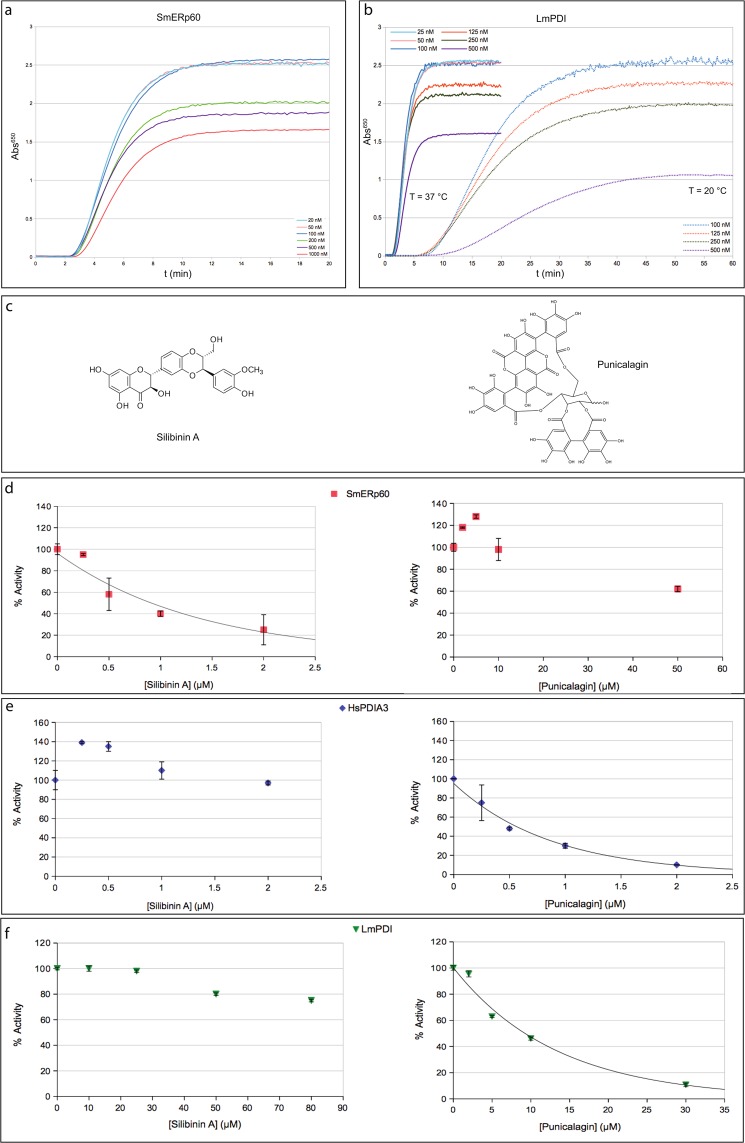
Redox activity of the recombinant PDIs and effect of selected natural products. Panel (a) Redox activity of SmERp60 measured at 37 °C with the turbidimetric assay. The reaction mixture contained SmERp60 at different concentrations (20, 50, 100, 200, 500 nM and 1 μM) and 130 μM insulin. The precipitation of insulin after S-S reduction was followed at 650 nm in a Cary 60 spectrophotometer (Agilent), see methods for details on the protocol. Panel (b) Redox activity of LmPDI measured with the turbidimetric assay. The assay mixture contained LmPDI at different concentrations (25, 50, 100, 125, 250 and 500 nM), 130 μM insulin. The precipitation of insulin after S-S reduction was followed at 650 nm and at two temperatures, 20 and 37 °C. Panel (c) Chemical structures of silibinin A (left) and punicalagin (right). Panel (d) Effect of selected natural products on the reduction of di-eosin oxidised glutathione by SmERp60, LmPDI and the host HsPDIA3/ERp57, followed by fluorimetry. The data are the average of 4 measurements. Redox activity of SmERp60 (50 nM), expressed as a % of reduction of di-eosinGSSG, in the presence of increasing concentrations of silibinin A (left, 0–2 μM) and punicalagin (right, 0–50 μM). Panel (e) Redox activity of host HsPDIA3/ERp57 (50 nM), expressed as a % of reduction of di-eosinGSSG, in the presence of increasing concentrations of silibinin A (left, 0–2 μM) and punicalagin (right, 0–2 μM). Panel (f) Redox activity of LmPDI (50 nM), expressed as a % of reduction of di-eosinGSSG, in the presence of increasing concentrations of silibinin A (left, 0–80 μM) and punicalagin (right, 0– 30 μM).

The ability of both recombinant proteins to reduce disulfide bonds was assayed using the classic turbidity test, based on the precipitation of reduced insulin chains^37^ (Fig. 3a,b) and also measured by fluorescence spectroscopy using di-eosin oxidised glutathione (di-eosin-GSSG) as substrate (Supplementary Fig. S5). The rate of reduction was 11.6 s^−1^ for SmERp60 and 24.3 s^−1^ for LmPDI, values in the same range of the human enzyme (16.8 s^−1^ for HsPDIA3/ERp57).

In the turbidity test, both freshly prepared SmERp60 and LmPDI were active at the concentrations tested (20–500 nM). At 20 °C and 37 °C SmERp60 had a lag time of 150 s and a rate constant of 0.46 ΔA_600_ min^−1^; the reaction was both temperature- and concentration- independent. On the other hand, LmPDI redox activity was indeed concentration-independent, but the slope at 20 °C was 0.22 ΔA_600_ min^−1^, with a lag time of 450 s, while at 37 °C the slope was 0.67 ΔA_600_ min^−1^, with a lag time of 90 s. As a control, *S*. *mansoni* Trx (SmTrx) at 20 °C and 100 nM was able to start the reaction after 100 s with a slope of 0.35 ΔA_600_ min^−1^, as previously reported^38^.

However, LmPDI was inactivated after one cycle of freeze-thawing, but it was able to regain activity when stored at 4 °C and incubated at 37 °C, prior to the experiment. In contrast, SmERp60 remained enzymatically active for 3 weeks at 4 °C and after three freeze-thaw cycles, unfortunately its inactivation was irreversible.

The purification in high yield of both parasite proteins highlighted a behaviour previously not reported. The insulin reduction assay is usually performed with PDI concentrations ranging between 10 and 100 nM, independently if the proteins are recombinant or native. In our work, we managed to raise the concentration in the assay up to 500 nM for both proteins and to 1 μM for SmERp60. Starting from 120–200 nM neither LmPDI nor SmERp60 were able to fully precipitate the insulin, always present in the assay in large excess (Fig. 3a,b). The prevention of precipitation of misfolded proteins is a feature shared by the holdases, ATP-independent chaperones, which are able to keep in quarantine misfolded proteins, avoiding aggregation^39^. A role of PDIs in the oxidative folding/refolding has already been reported in the literature [see^16,17,40,41^ for reviews], but a concentration dependent switch of function, from redox to chaperone, is a novelty. It is worth to note that PDIs are abundant in the parasites secretomes, therefore we may speculate that such a switch of function may play a role in the infection. Moreover we found that bacitracin, a widely used inhibitor of PDIs, with a controversial mechanism of action^42^, was able to inhibit the holdase function and to restore the oxidative power of both SmERp60 and LmPDI (Supplementary Fig. S6). In fact, when bacitracin (0.5 to 2 mM) was incubated with 500 nM SmERp60 and LmPDI, the precipitation of insulin started with a shorter lag time (more evident in SmERp60 than LmPDI) and the total absorbance went back to about 75–80% of the values obtained when the PDIs were used at lower concentrations (20–100 nM) (Fig. 3a,b *vs* Fig. S6).

Given that in *Caenorabditis elegans* and in *Dirofilaria immitis*, respectively a non parasitic and a parasitic worm, PDIs were reported to have a transglutaminase (TGase) activity^43,44^, we checked for this possibility, but no TGase activity was detected on either proteins, using a commercial colorimetric assay (TG2-CovTest, Covalab, Villeurbanne, France).

### Identification of binding partners of LmPDI and SmERp60 by SPR imaging

LmPDI and SmERp60 were circulated over gold affinity chips on which 76 ECM components and secreted macromolecules were spotted (the entire list is given in Supplementary Table S1). The identified partners of both PDIs are shown in Table 3. None of parasites PDI bound to proteoglycans or to collagens I to VI (neither native nor denatured), fibronectin, laminin-111, ECM-1 protein, thrombospondin-1, and vitronectin. In contrast, both proteins bound to heparan sulfate (HS). LmPDI bound also hyaluronan (HA) and SmERp60 interacted with tropoelastin, plasminogen, the GAG chondroitin sulfate (CS), and the ectodomain of the tumor endothelial marker 8 (TEM-8), also known as anthrax receptor 1. To further investigate the complex formed by SmERp60 and HS, we have performed SEC-SAXS on SmERp60 alone and in the presence of HS. Indeed a shift of the elution peak of the complex towards molecular mass compatible with a 1:1 stoichiometry was measured; the envelope derived from the corresponding scattering curve (Supplementary Fig. S3) was modelled with Gasbor on the ATSAS online server (https://www.embl-hamburg.de/biosaxs/atsas-online/gasbor.php). The structure of HsPDIA3/ERp57 was docked into the envelope leaving enough density for the GAG to be modelled inside (Supplementary Fig. S3). The same procedure was repeated with LmPDI in complex with HS and HA (Supplementary Fig. S2), here as well a shift in the elution peak was assigned to the complex formation in a *bona fide* 1:1 stoichiometry. It is interesting to note the change in the Kratky plot of the extrapolated scattering curves of the LmPDI-GAG complexes with respect to the one of LmPDI alone: there is a shift from a compact globular form to a folded, but more elongated particle, due to the elongated shape of the GAGs (Supplementary Fig. S4).

**Table 3:** Interactions of human ECM components with recombinant purified LmPDI and SmERp60.

	Binding partners of SmERp60	Binding partners of LmPDI
**Glycosaminoglycans**		
Chondroitin Sulfate (CS)	YES	no
Heparan Sulfate (HS)	YES	YES
Hyaluronan (HA)	no	YES
**Secreted proteins**		
Plasminogen	YES	no
**ECM proteins**		
Tropoelastin	YES	no
**Membrane receptors**		
Tumor endothelial marker 8 (TEM8)	YES	no

The binding was confirmed in two independent SPRi experiments for each protein; each ECM component was spotted 3 times on each chip (see methods and supplementary material).

### Natural compounds screening

Given the very limited number of effective drugs against schistosomiasis and leishmaniasis, and given that LmPDIs and SmErp60 are drug and/or vaccine targets^4,11^, we took the opportunity to screen a subset of a natural compounds library composed by antioxidant flavonoids, already used to search for inhibitors of HsPDIA3/ERp57^45–47^, which is over expressed in several cancers and is a drug target^48,49^. The rationale was to find reversible ligands, possibly able to discriminate between the host and the parasite PDIs. Among the compounds able to bind HsPDIA3/ERp57, two tannin derivatives attracted our attention: silibinin A and punicalagin (Fig. 3c). The first one is able to inhibit the redox activity of SmERp60 (IC_50_ = 750 nM), but not that of HsPDIA3/ERp57, nor of LmPDI; the second showed the exact opposite behaviour (Figs 3d *vs* 3e,f). Interestingly, silibinin A was previously reported to bind the catalytic *a/a*′ domains of HsPDIA3 with a Kd = 300 nM and to enhance the internalization of the protein-silibinin A complex in HeLa cells, thus affecting the non-redox functions of HsPDIA3/ERp57^46^. Moreover, silibinin A has been reported to have anti-angiogenic properties by targeting endothelial cells in the tumor microenvironment^50^. Punicalagin inhibits both HsPDIA3/ERp57 and LmPDI, but the structural differences result in dramatically different IC_50_, which is 0.5 μM for the host HsPDIA3/ERp57 and 7.3 μM for LmPDI. Therefore, while Silibinin A might be a good starting point to design selective anti-helminthic drugs, punicalagin might only be used in cases when the host PDI is deregulated, not when the host is infected by *Leishmania*.

## Discussion

*Leishmania major* is one of the causative agents of cutaneous leishmaniasis; it is an intracellular eukaryotic parasite targeting macrophages of the dermal tissues. *Schistosoma mansoni* is the widest spread agent of intestinal schistosomiasis; it is a blood feeder extracellular trematode, colonizing the mesenteric veins around the liver. PDIs are widespread and highly conserved proteins mainly dedicated to oxidative folding and signalling. Despite architectural conservation, the differences in PDI’s sequences might result in significant changes in dynamics and protein-protein interactions. This is especially true for the two proteins examined in this work and chosen because of their abundance in the secretomes of *L*. *major* and *S*. *mansoni*. In fact, as can be appreciated from the sequence alignment, the catalytically active Trx-like domains are well conserved among the PDIs from *H*. *sapiens* and both parasites, but the central redox-inactive Trx-like domains are less conserved (Fig. 1a). Moreover, the connections between the four domains differ in length between SmERp60 and LmPDI. This results in the latter assuming a compact structure, more reminiscent of the yeast PDI, as proved by SAXS, and SmERp60 being a flexible monomer, with at least three main conformations, deconvolved from SAXS data, similarly to the host homologues HsPDIA3/ERp57 (PDB 3F8U^33^) and HsPDIA1 (PDB 4EKZ, 4ELI^36^) [see also^17^ for a review].

Recombinant SmERp60 is more stable than LmPDI to freeze-thaw cycles and does not display a temperature-driven function nor inactivation. This might reflect the fact that Schistosomes are adapted to environments with different temperatures: a mammalian host, a freshwater snail and freshwaters of tropical rivers/lakes.

Both recombinant PDIs show a previously undisclosed concentration-dependent switch of function from redox enzymes to holdase chaperones, indeed an example of moonlighting behaviour. Another secreted protein from *S*. *mansoni* showed this same behaviour, the thioredoxin peroxidase, SmPrxI, which looses its redox activity and acquires a holdase one at high concentration^51^. It will be interesting to enlarge the study to other redox proteins secreted by the parasite.

The screening of natural compounds has highlighted silibinin A as putative lead compound able to inhibit SmERp60, but not the host counterpart. It will be interesting to investigate its role *in vivo* in animal models of infection. It is worth reminding that PDIs are both intracellular and secreted proteins, so putative inhibitors might be useful in two stages of the infection. A compound targeting intracellular PDIs might interfere with its redox balance (thiol/disulfide) and therefore increase the oxidative stress in the parasites. A compound targeting secreted PDIs might interfere with host-parasite interactions and possibly limit infection.

GAGs are binding partners of both parasite PDIs (Table 3), but their repertoire is different: LmPDI binds heparan sulfate (HS) and hyaluronan (HA), while SmERp60 binds HS and chondroitin sulfate (CS). HS is known to regulate inflammation^52,53^; HA is involved in macrophage recruitment in inflammation sites^54^. In addition HS and CS have immune-modulatory properties^55,56^. The interactions of GAGs with both PDI may facilitate their attachment to the host ECM and hence host invasion. It is known that GAGs displayed at the surface of macrophages are involved in *Leishmania* recognition and uptake^7,22^, we have shown by SPRi that LmPDI is one of the molecular interactors.

Schistosomes are blood feeders and live in the blood stream of the mesenteric veins, so it is not a surprise that SmERp60 binds human circulating plasminogen. At present only the secreted enolase from *S*. *japonicum* has been shown to bind human plasminogen^57^. SmERp60 also interacts with tropoelastin, in line with the fact that schistosomes are able to degrade elastin^58^. Interestingly, the protein interacts with Tumor Endothelial Marker 8 (TEM-8). This type I transmembrane receptor is known to be over-expressed in inflammatory processes and in cancer^59^; it is one of the receptors for the *Bacillus anthracis* protective antigen toxin and the Seneca Valley Virus^60,61^; it has also been shown to modulate lymphocyte maturation and ECM remodelling^62,63^. Thus, the interaction between SmERp60 and TEM-8 suggests either a role for this receptor in host-pathogen interaction or a doorway for the parasite to hijack the host immune system. Despite LmPDI does not bind TEM-8, intact live promastigotes of *L*. *major* were reported to strongly interact with it^23^, therefore the *Leishmania* ligand needs to be identified among other components of the secretome. Very intriguingly, while this manuscript was under revision, an *in silico* study on host-parasite interactions predicted SmERp60 to be one of the main hubs for *S*. *mansoni*^14^, unfortunately there were no data on *L*. *major*, but based on our results and on our previous study on whole promastigotes^23^, we may infer that LmPDI would be a player in host-parasite interactions as well.

In conclusion, LmPDI and SmERp60 are both monomeric and present a concentration-dependent switch of function from thiol-disulfide redox activity to holdase. They interact with GAGs known to regulate infection and immunity, thus these interactions may play a role in the host-parasite cross-talk. Finally, SmERp60 binds to TEM-8, which stimulates angiogenesis and serves as receptor for other pathogens; the role of this interaction in *S*. *mansoni* infection opens the way to further investigations.

## Methods Section

### Cloning expression and purification of *Leishmania major* Protein Disulfide Isomerase (LmPDI)

The gene encoding for LmPDI (Q8I8E1, unreviewed entry of UniProtKB) was cloned into pET30-a vector (Novagen) within the restriction sites *BamHI* and *EcoR*I, with a sequence coding for 6His-cleavable with enterokinase (FLAG) tag at the N-terminus, using standard molecular biology techniques^64^ and the primers listed in Supplementary Table S2. The vector was transformed into *E*. *coli* BL21(DE3) (New England Biolabs). Bacteria were grown at 37 °C in liquid LB medium (DIFCO) containing kanamycin (50 μg/ml, SIGMA) shaking at 180 rpm until Abs_600_ reached 0.6. The expression of the protein was induced overnight at 25 °C with 0.1 mM isopropyl-β-D-thio-galactopyranoside (IPTG, SIGMA). Bacteria were centrifuged 10 min at 5000 × *g* and the pellets frozen at −20 °C until use. The thawed pellets were resuspended in 50 mM Tris/HCl pH 7.4, 150 mM NaCl (TBS); then 3 U of DNase (SIGMA) and one tablet of protease inhibitors (Complete EDTA-free, Roche) was added to the resuspended pellets and incubated for 20 min on ice, before lysing the membranes by ultrasonication (Branson). Five to ten cycles of ultrasound at 40% amplitude with 5 seconds on and 10 s off pulses on ice were used to disrupt the cells. The lysed cells were then centrifuged at 4 °C for 50 min at 15000 × *g*. The supernatant was collected and the recombinant protein was purified by affinity chromatography on a Nickel-bound nitrilo-triacetic acid (Ni-NTA) agarose resin (Qiagen), equilibrated at 4 °C in TBS. The supernatant was loaded twice on the column, which was washed with 1 column volume of TBS containing 20 mM imidazole and then elution was performed with a gradient of imidazole from 20 to 500 mM in TBS. The protein eluted when the concentration of imidazole was around 200 mM and the imidazole was eliminated by dialysis in TBS, prior to protein concentration. The purity of the protein was checked by SDS-PAGE (Supplementary Fig. S7), its identity by Western Blot (mouse monoclonal Anti-Flag Ab, F3165 SIGMA) and the protein was concentrated by ultrafiltration (Vivaspin 10-kDa cutoff, Sartorius) up to 2 mg/ml and stored at 4 °C until use. The His-FLAG tag was cleaved by incubation with enterokinase (11351311001, Roche) 4 h at 20 °C and the cleaved protein was purified with an inverse affinity chromatography on Ni-NTA-agarose resin (Supplementary Fig. S7).

### Cloning expression and purification of *Schistosoma mansoni* Protein disulfide isomerase (SmERp60)

The sequence coding for SmERp60 (UniProtKB P38658) was cloned into pGEX4T-1 (GE-Healthcare) within the restriction sites *Bam*HI and *Xho*I, after the thrombin recognition site, using standard molecular biology techniques^64^. The plasmid was inserted into *E*. *coli* DH5α competent cells (New England Biolabs) by thermal shock. The transformed cells were grown in LB medium containing 100 μg/ml ampicillin (SIGMA) at 37 °C shaking at 180 rpm until A_600_ reached 0.8. Expression was then induced with 0.1 mM IPTG at 20 °C overnight. Cells were harvested by centrifuge for 10 min at 5000 × *g* and resuspended into TBS. In order to purify the recombinant protein, the cells, after the addition of 3 U DNase (SIGMA) and 1 tablet of protease inhibitor (Complete EDTA-free, Roche), were disrupted by ultrasound (on ice with 5 s pluses on and 10 s off, 40% amplitude on a Branson instrument) and centrifuged 1 h at 15000 × *g* and 4 °C. The resulting supernatant was applied to glutathione-sepharose beads (GE-Healthcare), previously equilibrated with TBS buffer, and recirculated three times. The beads were then washed with 40 volumes of TBS and bovine thrombin [SIGMA, T4648] was added to the slurry overnight in order to cleave the GST tag. The eluate was collected, passed onto a benzamidine column (equilibrated in TBS) to remove the thrombin and concentrated up to 8 mg/ml by ultrafiltration (Vivaspin 10 kDa cutoff, Sartorius). The protein purity was assessed by SDS-PAGE (Supplementary Fig. S7).

### Circular dichroism

Both purified proteins were dialysed *vs* 10 mM potassium phosphate buffer pH 7.0 before collecting seven CD spectra for each protein between 185 and 280 nm at 20 °C on a Jasco J-810 instrument. The averaged spectra, after buffer subtraction, were deconvolved and analysed on the DichroWeb website^65^. The self-consistent method for analyzing protein CD spectra, SELCON3, and CD secondary structure programme, CDSSTR^66^, were used to estimate the percentage of secondary structure of recombinant PDIs (Table 1), by using the reference dataset sp175.

### Identification of PDI interacting macromolecules by SPRi

SPR binding assays were performed in a Biacore Flexchip system (GE Healthcare, Facility of UMS 3444/US8, Lyon, France). Proteins (50–200 μg/ml) and GAGs (0.5–1 mg/ml) were spotted directly in triplicate onto the gold surface of a Gold Affinity chip (GE Healthcare) using a non-contact PiezoArray spotter (Perkin-Elmer Life Sciences) as previously described^23,67^. The spotted chip comprised 76 biomolecules and controls (buffers and tags) (see Supplementary Table S1 for a complete list). The chip was blocked with a buffer containing mammalian proteins (Flexchip blocking buffer, GE Healthcare) for 5 times for 5 minutes each. The blocked chip was then equilibrated with 10 mM Hepes pH 7.4, 150 mM NaCl, 0.05% Tween 20 (HBS-T) at 500 μl/min for 90 min. LmPDI and SmERp60 were diluted in HBS-T at a final concentration of 500 nM and injected over the chip surface at 25 °C for 25 min at the same flow rate. The dissociation of the complexes was monitored in HBS-T flow for 40 min. Data collected from reference spots (gold surface) and from buffer spots were subtracted from those collected on spotted proteins or GAGs to obtain specific binding curves. The binding was confirmed in two independent SPRi experiments for each recombinant protein. Since each biomolecule was spotted in triplicate on each chip, the results were the average of six sensorgrams; any given interaction positive for >4/6 was considered true.

### Disulfide reductase activity assays

The capability of SmERp60 and LmPDI to reduce disulfide bonds was assessed by two methods: a standard turbidimetric assay using insulin as a substrate^37^; and a fluorimetric assay using oxidised glutathione conjugated with eosin (di-eosin-GSSG^45^).

Insulin (I6634, SIGMA) was dissolved in 20 mM Tris/HCl buffer pH 8. The solution was then acidified to pH 2–3 and quickly titrated back to pH 8. This procedure leads to the cleavage of the polypeptide into two chains, linked by a disulphide bond. The reduction of this disulphide bridge causes the separation of the two insulin chains and their precipitation, with consequent increase in the turbidity of the solution that can be monitored by measuring the absorbance at 650 nm on a Cary 60 spectrophotometer (Agilent). The assay mixture contained 0.1 M potassium phosphate buffer pH 7.0, 1 mM EDTA, 130 μM insulin, 500 μM dithiothreitol (DTT) and varying concentrations of SmERp60 and LmPDI ranging from 20 to 500 nM. A control reaction was performed by omitting the enzyme. All the assays were performed at 25 °C and at 37 °C. *S*. *mansoni* thioredoxin (SmTrx) was used as a positive enzyme control^38^.

The fluorescent probe di-eosin-GSSG (λ_exc_ = 520 nm; λ_em_ = 545 nm) was used as alternative to turbidimetry to check the reduction of disulphide bridges. To produce the probe, 1 ml of GSSG (SIGMA) 0.1 mM was incubated with 1 mM eosin-5-isothiocyanate (SIGMA) in 100 mM potassium phosphate, 0.2 mM EDTA pH 8.8, for 1 h at room temperature. The reaction mixture was purified on a G-25 column equilibrated with 100 mM potassium phosphate, 0.2 mM EDTA pH 7.0. The elution was monitored by measuring the absorbance at 525 nm and the concentration of di-eosin-GSSG was calculated by using the extinction coefficient ε_525_ = 88000 M^−1^cm^−1^. The assay mixture contained 50 nM protein (HsERp57/PDIA3, SmERp60, LmPDI), 200 nM di-eosin-GSSG, 5 μM DTT in PBS 1X, 0.2 mM EDTA pH 7.4. For the inhibition studies, increasing concentrations (0.2–50 μM) of punicalagin and silibinin A (Sapienza collection of natural compounds, Sapienza University of Rome, Italy) were added to the above reaction mixture and the fluorescence quenching was monitored at 545 nm (λ_exc_ = 520 nm) on a Jasco FP8500 spectrofluorimeter.

### Structural analysis by Small Angle X-ray Scattering (SAXS)

Data collection for the SAXS studies was initially performed at the BioSAXS beamline P12 of PETRAIII (Hamburg, Germany) using serial dilutions of SmERp60 from 6 to 0.5 mg/ml and of LmPDI from 2 to 0.5 mg/ml. Further analysis on new batches of purification was then performed at BioSAXS beamline BM29 of ESRF (Grenoble, France)^68^, where the wavelength was 0.9998 Å and the sample to detector distance 2.8 m. Size exclusion chromatography was performed on a HPLC system (Shimadzu) in line with BM29 beamline (SEC-SAXS). Several samples concentrated from 4 to 10 mg/ml were automatically injected (50 μl) into a Superdex S200 5/150 (GE Healthcare), previously equilibrated with TBS. The column was run at 20 °C with a constant flow of 0.2 ml/min and 1 image/s was collected. The scattering images were collected on a Pilatus 1 M detector (Dectris) and converted into 1d profiles with BsXCube; the scattering profiles were analysed off-line using the suite ATSAS^69^. Scattering of the buffer was subtracted as background from the protein measurements with PRIMUS. HPLC-SAXS data were analysed with CHROMIXS^70^ and US-SOMO^71^. The evaluation of the radius of gyration (Rg) and the forward scattering intensity (I(0)) were performed using the Guinier approximation. *Ab initio* analysis was performed either locally with DAMMIF/DAMMIN^72^ or online with GASBOR (https://www.embl-hamburg.de/biosaxs/atsas-online/gasbor.php); validation and averaging was performed with DAMAVER^73^; rigid body modelling of known structures into SAXS envelope was performed with SUPCOMB^69^. Flexibility was checked with EOM using the default parameters (10000 initial native-like models and constant subtraction allowed)^34^; the final selection retained 3 final structures, built starting from the *a*, *a*′, *b-b*′ modules of HsPDIA3/ERp57 and HsPDIA1. The model intensities were back computed with CRYSOL^74^ and 3D graphics were produced with CCP4MG^75^.

## Supplementary information

Supplementary Information
