# Supplementary material for: A comparative analysis of secreted protein disulfide isomerases from the tropical co-endemic parasites Schistosoma mansoni and Leishmania major
Source: Sci Rep. 2019 Jul 2;9:9568. doi: 10.1038/s41598-019-45709-8 (PMC6606611; doi:10.1038/s41598-019-45709-8)

Supplementary Material

A comparative analysis of protein disulfide isomerases from the tropical co-endemic parasites *Schistosoma mansoni* and *Leishmania major*

Adriana E. Miele,[a,b] Sofiane Badaoui,[b] Lorenzo Maugliani,[a,b] Romain Salza,[b] Giovanna Boumis,[a] Silvia Chichiarelli,[a] Bertrand Duclos,[b] Sylvie Ricard-Blum,[b]

[a] Dept. Biochemical Sciences, Sapienza University of Rome, P.le Aldo Moro 5, Rome, 00185, Italy;
[b] ICBMS UMR 5246, CNRS - Université de Lyon, 43 Boulevard du 11 Novembre 1918, Villeurbanne cedex, 69622, France.

Supplementary Table S1. List of biomolecules spotted on the SPRi chip.
Supplementary Table S2. List of DNA primers used for cloning SmERp60 and LmPDI.
Supplementary Figure S1. SAXS data of LmPDI at 5°C.
Supplementary Figure S2. SEC-SAXS of LmPDI and of the complexes LmPDI-HA, LmPDI-HS at 20 °C.
Supplementary Figure S3. SAXS of the complex SmERp60-HS at 20°C.
Supplementary Figure S4. Normalised Kratky plot of LmPDI and of the complexes LmPDI-HA, LmPDI-HS.
Supplementary Figure S5. Kinetics of di-eosin-GSSG reduction by PDIs.
Supplementary Figure S6. Kinetics of insulin reduction by PDIs in presence of bacitracin.
Supplementary Figure S7. Analysis by SDS PAGE of purified SmERp60 and LmPDI.

Supplementary Table S1. List of biomolecules tested in binding assays performed by Surface Plasmon Resonance (SPR) imaging. ChEBI: Chemical Entities of Biological Interest, CPX: Complex Portal identifier, PRO_features are from UniProtKB.

**Biomolecules spotted on the chip**	**Identifiers (ChEBI, UniProtKB or Complex Portal)**[1]	**Source (commercial or PMID)**
Aggrecan	P37840	Sigma-Aldrich, A1960
Anastellin (Fibronectin fragment III_1-C)	P02751 (PRO_0000390479, 631-702)	Sigma-Aldrich, F3542
Angiopoietin-like protein 4 (AngPTL-4)	Q9BY76 (26-406)	R&D Systems, 4487-AN
β-2 microglobulin	P61769	Sigma-Aldrich, M4890
Biglycan	P21809	Sigma-Aldrich, B8041
Brevican	Q96GW7 (23-911)	R&D Systems, 4009-BC
Bovine serum albumin (BSA)	P02769	Sigma-Aldrich, A2153
Adiponectin, complement C1q containing protein	Q15848	Sigma-Aldrich, SRP4901
Carboxymethyl-dextran (CM-D)	ChEBI:52071	Sigma-Aldrich, 86524
Collagen I	CPX-1650	Sigma-Aldrich, C7774
Collagen II	CPX-3105	Sigma-Aldrich, C1188
Collagen III	CPX-1714	Sigma-Aldrich, C4407
Collagen IV	CPX-1723	Sigma-Aldrich, C7521
Collagen V	CPX-1727	Sigma-Aldrich, C3657
Collagen VI	CPX-1736	GeneTex, GTX27538
Collagen VI (vWF1 domain)	P12109 (20-256)	Expressed in Ricard-Blum's laboratory (PMID 24117177)
Collagen XVIII (NC1 domain)	CPX-1759 (1443-1754)	Expressed in Ricard-Blum's laboratory (PMID: 14585835, 19542224, 19502598, 24478075, 24117177, 28106549); transfected HEK cells provided by Prof. B. Olsen (Harvard Medical School, USA)
C1q, complement component	P02745	Generous gift of Dr N.M. Thielens (Univ. Grenoble Alps,

[1] Whenever a fragment has been used, the numbers in parenthesis indicate the first and last amino acid.

		France) PMID 40870
Chondroitin sulfate (CS)	CHEBI:37397	Sigma-Aldrich, C8529
Dermatan sulfate (DS)	CHEBI:18376	Sigma-Aldrich, C3788
Endostatin (ES, expressed in *Pichia pastoris*)	P39060 (PRO_0000005794)	Sigma-Aldrich, E8154
ES (expressed in HEK293 cells)	P39060 (PRO_0000005794)	Expressed in Ricard-Blum's laboratory (PMID: 14585835, 19542224, 19502598, 24117177, 24478075, 28106549, DOI: 10.1039/9781788010283-00398); transfected HEK cells provided by Prof. B. Olsen (Harvard Medical School, USA)
ES mutant D104N	P39060 (PRO_0000005794)	Expressed in Ricard-Blum's laboratory (PMID: 19502598)
Fibrinogen	P02671-75-79 (alpha, beta, gamma)	Sigma-Adrich, F3879
Fibromodulin	P13605	Sigma-Aldrich, F6921
Fibronectin (FN)	P02751	Sigma-Aldrich, F2006
Fibronectin digested with α-chymotrypsin	P02751	Sigma-Aldrich, F2006
H-ficolin (Ficolin-3)	O75636	Generous gift of Dr N.M. Thielens, Univ. Grenoble Alps, France (PMID 19109177)
L-ficolin (Ficolin-2)	Q15485	Generous gift of Dr N.M. Thielens, Univ. Grenoble Alps, France (PMID 19109177)
M-ficolin (Ficolin-1)	O00602	Generous gift of Dr N.M. Thielens, Univ. Grenoble Alps, France (PMID 20032467)
Glypican 1 ectodomain (ED)	P35052 (24-530)	R&D Systems, 4519-GP
Glypican 2 ED	Q8N158 (18-553)	R&D Systems, 2304-GP
Glypican 3 ED	P51654	R&D Systems, 2119-GP
Glypican 5 ED	P78333 (25-554)	R&D Systems, 2607-G5
Glypican 6 ED	Q9Y625 (24-355)	R&D Systems, 2845-GP
Hyaluronic acid (HA)	CHEBI:16336	Acros Organics, 25177
HA (25-75 kDa)	CHEBI:16336	Sigma-Aldrich, S0326

Heparin (HP, high molecular weight)	CHEBI:28304	Sigma-Aldrich, H3393
HP (low molecular weight, 3 kDa)	CHEBI:28304	Sigma-Aldrich, H3400
HP 2-O-desulphated	CHEBI:28304	Iduron, DSH001/2
HP 6-O-desulphated	CHEBI:28304	Iduron, DSH002/6
HP N-desulphated-re-N-acetylated	CHEBI:28304	Iduron, DSH004/Nac
Heparan sulfate (HS, bovine)	CHEBI:28815	Sigma-Aldrich, H7640
HS (pig)	CHEBI:28815	Celsus Lab, HO-3105
Human extracellular matrix protein 1 (hECM1)	Q16610 (20-540)	R&D Systems, 3937-EC
Integrin α4β1 ectodomain (ED)	CPX-1802	R&D Systems, 5668-A4
Integrin α5β1 ED	CPX-1794	R&D Systems, 3230-A5
Integrin αvβ3 ED	CPX-1795	R&D Systems, 3050-AV
Integrin αvβ5 ED	CPX-1796	R&D Systems, 2528-AV
Laminin-111	CPX-3008	Sigma-Aldrich, L2020
Lumican	P51884 (19-338)	R&D Systems, 2846-LU
Neurocan	P55066 (23-637)	R&D Systems, 5800-NC
Neuroglycan C	O95196 (31-420)	R&D Systems, 5685-NG
Osteopontin (OSP) with BSA	P10451	Sigma-Aldrich, O4264
Osteonectin (SPARC)	P09486	Immundiagnostik AG, A4225AG.1
Perlecan	Q05793	Sigma-Aldrich, H4777
Plasminogen	P00747	R&D Systems, 1939-SE
Syndecan 1 ectodomain (ED)	P18827 (18-251)	R&D Systems, 2780-SD
Syndecan 2-ED	P34741 (19-144)	R&D Systems, 2965-SD
Syndecan 3-ED	O75056 (48-383)	R&D Systems, 3539-SD
Syndecan 4-2	P31431 (19-145)	R&D Systems, 2918-SD
Tumor endotelial marker 8 (TEM 8)/Anthrax toxin receptor 1	Q9H6X2-2 (1-368)	Abnova, H00084168-P01
Transglutaminase-2 (TG-2, guinea	P08587	Sigma-Aldrich, T5398

pig)		
TG-2 human	P21980	Immundiagnostik AG, AK3010AG.1
Trombospondin-1 (TSP-1)	CPX-1785	Immundiagnostik AG, AW1011AG.1
Tropoelastin	P15502	Sigma-Aldrich, T0706
Tropomyosin (pig)	P42639	Sigma-Aldrich, T2400
Vascular endothelial growth factor (VEGF)	CPX-1977	Sigma-Aldrich, V7259
VEGF receptor-2 (VEGFR-2)	P35968 (20-764)	R&D Systems, 357-KD
Vitronectin	P04004	R&D Systems, 2349-VN

As a control, the following buffers were also spotted on the chip, since most of the commercial products were lyophilised in different buffers: 10 mM Hepes pH 7.4, 150 mM NaCl (HBS); HBS + 1 mM $ZnCl_2$; PBS pH 7.4 (10 mM Na/K phosphate, 137 mM NaCl, 2.7 mM KCl); TBS pH 7.4 (50 mM Tris/HCl, 150 mM NaCl); TBS + 1 mM $CaCl_2$; 50 mM Tris/HCl pH 7.4; Flexchip Blocking buffer (GE Healthcare, BR-1007-08).
The tags of LmPDI and SmERp60 were also immobilised, as a control: FLAG peptide (200 µg/ml) in HBS; glutathione S-transferase (GST) in HBS (100 µg/ml).

References:

PMID 40870: Arlaud GJ, Sim RB, Duplaa AM, Colomb MG. Differential elution of Clq, Clr and Cls from human Complement1 bound to immune aggregates. Use in the rapid purification of Cl subcomponents. Mol Immunol. 1979 Jul;16(7):445-50.

PMID 14585835: Ricard-Blum S, Féraud O, Lortat-Jacob H, Rencurosi A, Fukai N, Dkhissi F, Vittet D, Imberty A, Olsen BR, van der Rest M. Characterization of endostatin binding to heparin and heparan sulfate by surface plasmon resonance and molecular modeling: role of divalent cations. J Biol Chem. 2004 Jan 23;279(4):2927-36.

PMID 19109177: Lacroix M, Dumestre-Pérard C, Schoehn G, Houen G, Cesbron JY, Arlaud GJ, Thielens NM. Residue Lys57 in the collagen-like region of human L-ficolin and its counterpart Lys47 in H-ficolin play a key role in the interaction with the mannan-binding lectin-associated serine proteases and the collectin receptor calreticulin. J Immunol. 2009 Jan 1;182(1):456-65.

PMID 19502598: Faye C, Moreau C, Chautard E, Jetne R, Fukai N, Ruggiero F, Humphries MJ, Olsen BR, Ricard-Blum S. Molecular interplay between endostatin, integrins, and heparan sulfate. J Biol Chem. 2009 Aug 14;284(33):22029-40.

PMID 19542224: Faye C, Chautard E, Olsen BR, Ricard-Blum S. The first draft of the endostatin interaction network. J Biol Chem. 2009 Aug 14;284(33):22041-7.

PMID 20032467: Gout E, Garlatti V, Smith DF, Lacroix M, Dumestre-Pérard C, Lunardi T, Martin L, Cesbron JY, Arlaud GJ, Gaboriaud C, Thielens NM. Carbohydrate recognition properties of human ficolins: glycan array screening reveals the sialic acid binding specificity of M-ficolin. J Biol Chem. 2010 Feb 26;285(9):6612-22.

PMID 24117177: Salza R, Peysselon F, Chautard E, Faye C, Moschcovich L, Weiss T, Perrin-Cocon L, Lotteau V, Kessler E, Ricard-Blum S. Extended interaction network of procollagen C-proteinase enhancer-1 in the extracellular matrix. Biochem J. 2014 Jan 1;457(1):137-49.

PMID 24478075: Fatoux-Ardore M, Peysselon F, Weiss A, Bastien P, Pratlong F, Ricard-Blum S. Large-scale investigation of *Leishmania* interaction networks with host extracellular matrix by surface plasmon resonance imaging. Infect Immun. 2014 Feb;82(2):594-606.

PMID 28106549: Salza R, Lethias C, Ricard-Blum S. The Multimerization State of the Amyloid-β42 Amyloid Peptide Governs its Interaction Network with the Extracellular Matrix. J Alzheimers Dis. 2017;56(3):991-1005.

Vallet SD, Deddens L, Vonarburg A, Salza R., Faye C., Aranyos A., Thierry-Mieg N, Ricard-Blum S. Strategies for Building Protein-glycosaminoglycan interaction networks combining SPRi, SPR and BLI. Handbook of Surface Plasmon Resonance, Chapter 11, 2017; 398–414. Royal Society of Chemistry.

Supplementary Table S2. List of DNA primers used to clone LmPDI and SmERp60 into pET30a and pGEX4T-1, respectively. The sequences for the restriction enzymes used for cloning are bold and the stop codons are underlined.

**Primer's name**	**Primer's sequence**
BamHI-LmPDI *forward*	5' – CC**GGATCC**ATGGAGGTGCAGGTGGCCAC - 3'
EcoRI-LmPDI *reverse*	5' – CG**GAATCC**CTACAAATCTTCCTCTTCGCTG - 3'
BamHI-SmERp60 *forward*	5' - CC**GGATCC**AGCAAAGTTCTGGAACTGACC -3'
XhoI-SmERp60 *reverse*	5' – CG**CTCGAG**TTACAGTTCGGATTTTTTCGGG -3'

Supplementary Figure S1. Logarithmic and Kratky plots of X-ray scattering curves of LmPDI at 5 °C. The signal is the average of 10 curves, after buffer subtraction. 2D images collected at SWING (SOLEIL Synchrotron, St Aubin, France) with the sample changer under flow (75 µl/min); 1 second acquisition time; E = 12 keV; detector distance 2087.45 mm. Buffer TBS (50 mM Tris/HCl pH 7.4, 150 mM NaCl) was recirculated before and after every sample.

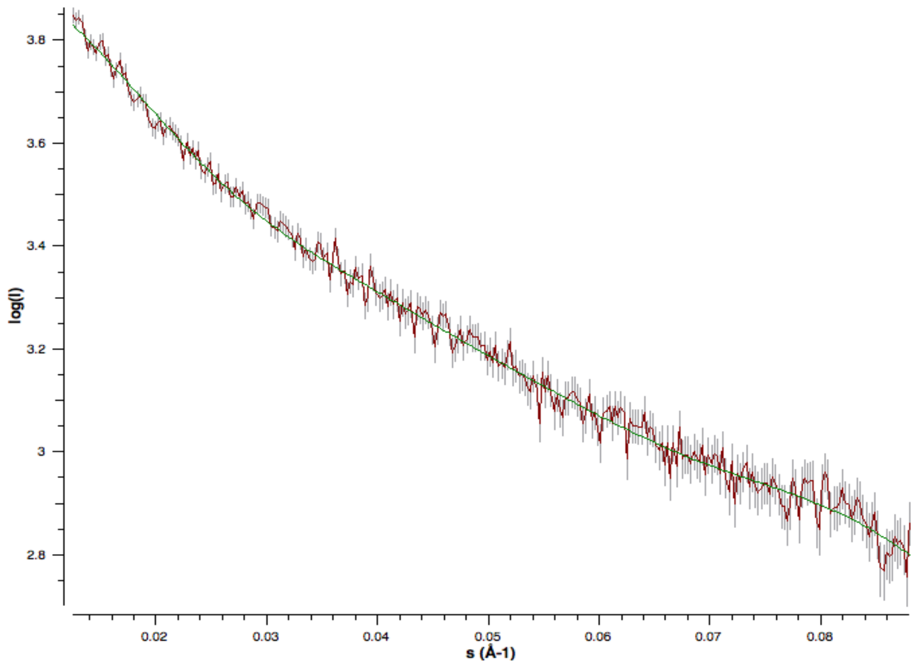

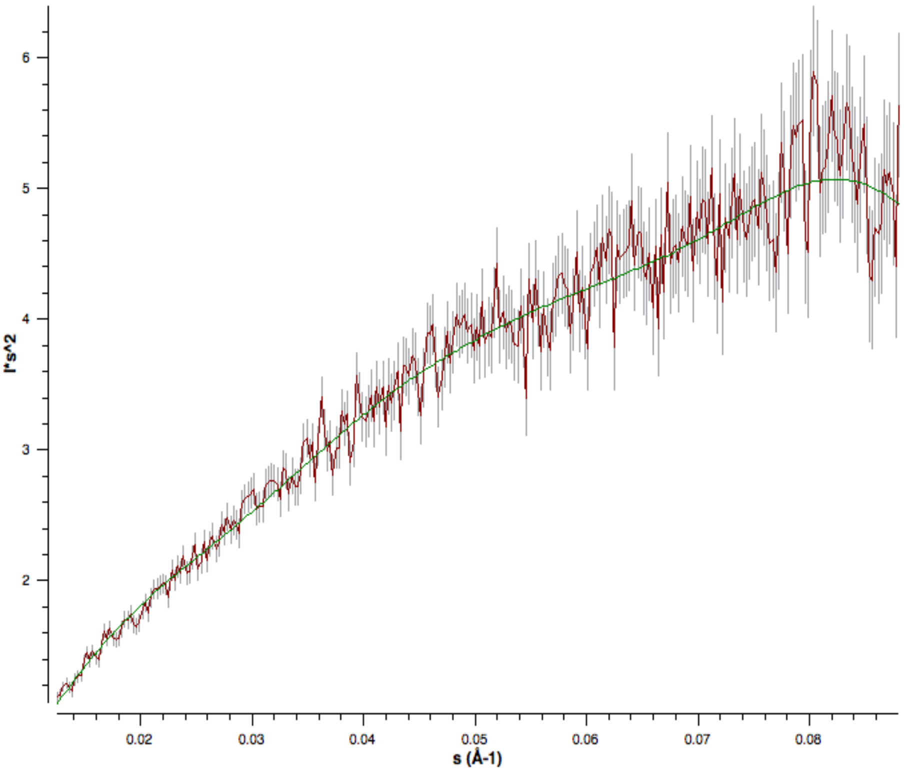

Supplementary Figure S2. Overlaid chromatograms of LmPDI alone (**green**), Lm-PDI in complex with hyaluronan (HA) (LmPDI-HA, **blue**) and with heparan sulfate (HS) (LmPDI-HS, **orange**) from three successive runs of HPLC (Shimadzu) size exclusion chromatography coupled to SAXS beamline BM29 (ESRF, Grenoble). Column Superdex S200 HR 5/150 (GE Healthcare), buffer TBS, flow 0.4 ml/min. The black triangles are the Rg values (nm) computed with US-SOMO HPLC-SAXS analysis (3.2 nm alone and 4.0 nm in complex). The maximum dimension of the particles (D_{max}), derived from the P(r) function, increases from 9.7 nm in the absence of ligand to 12 nm in the presence of either of the two GAGs.

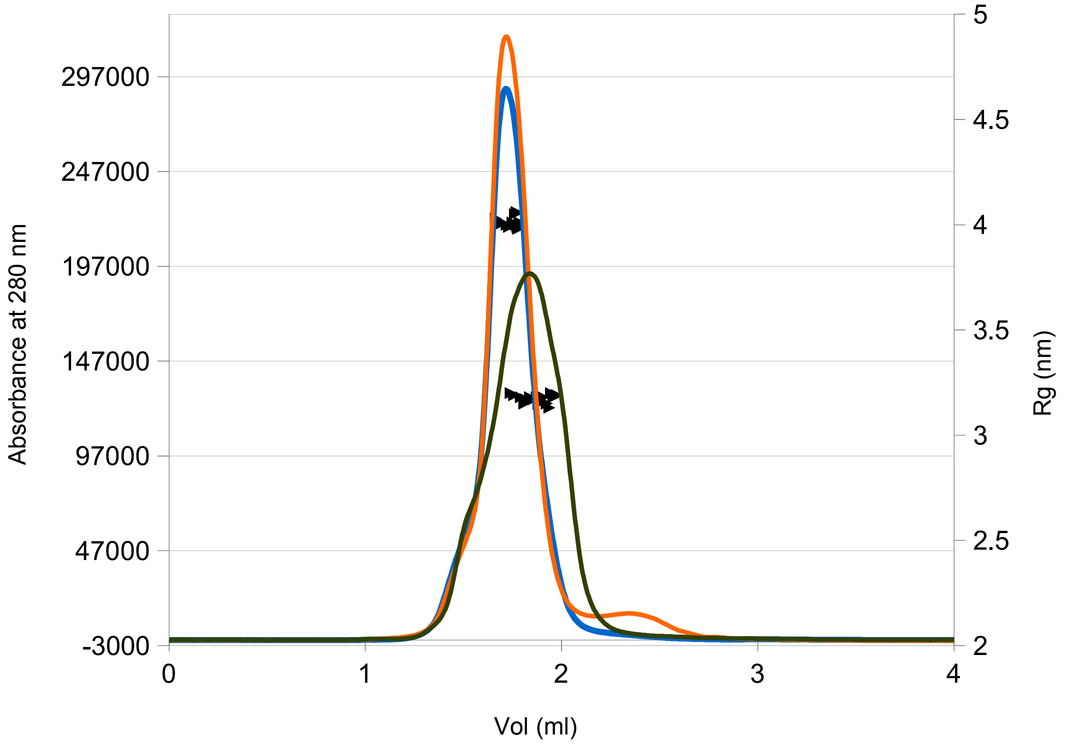

Vol (ml)

Supplementary Figure S3. Model of the complex between SmERp60 and heparan sulfate (HS). The *ab initio* model was produced with Gasbor on the ATSAS online server. The structure of HsERp57/PDIA3 was docked in the envelope with Supcomb. The figure was prepared with CCP4MG. The corresponding fit to the experimental data is presented below.

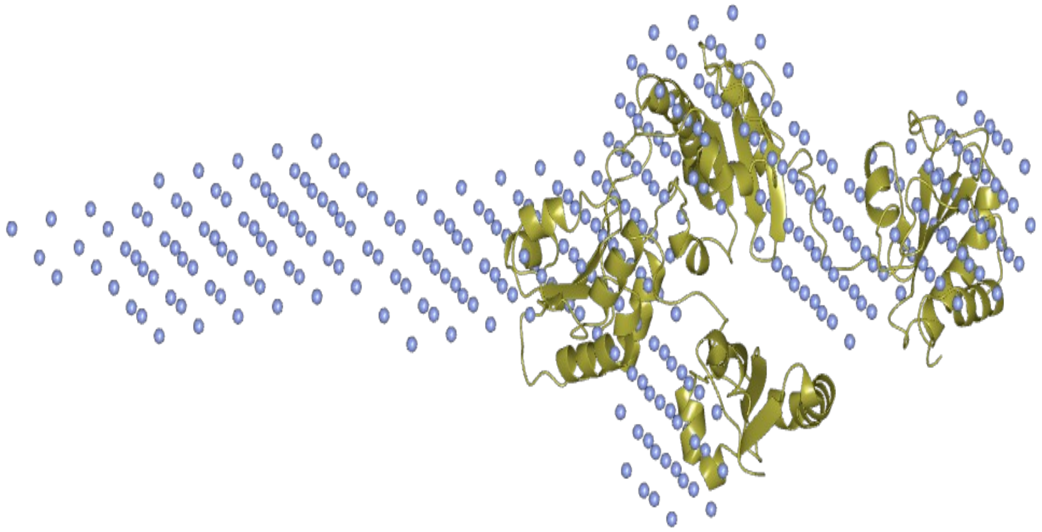

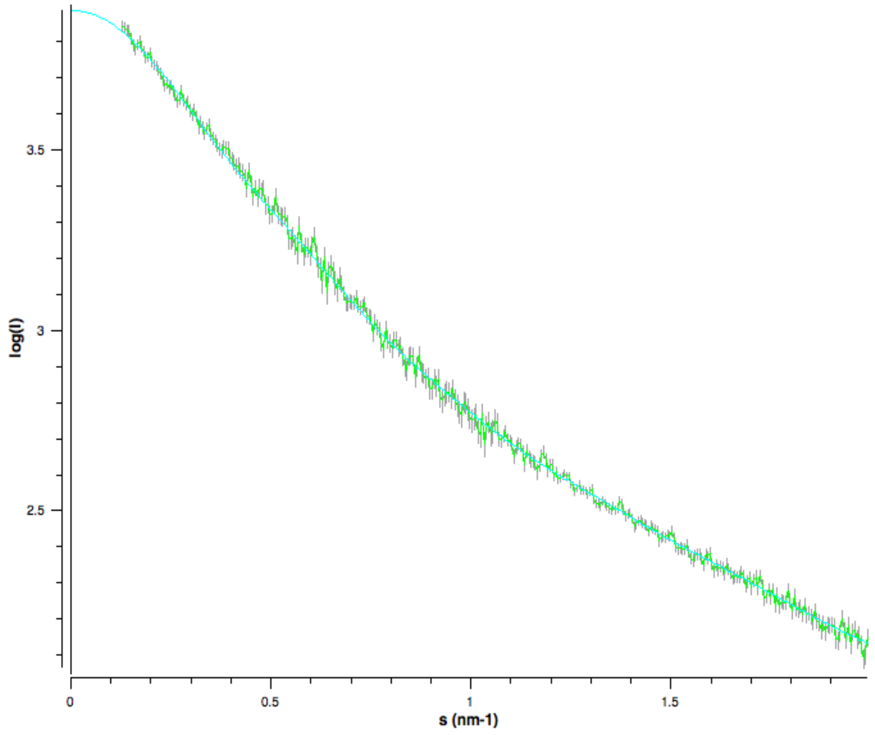

Supplementary Figure S4. Superimposed scattering curves extrapolated by US-SOMO HPLC-SAXS from the peaks of the three HPLC runs, in the normalised dimensionless Kratky representation. The curve of LmPDI is in green, the curve of the complex LmPDI-HA in blue and that of LmPDI-HS in orange. A transition from a compact to a more elongated particle is evident in this representation, which completes the quantitative analysis of **Figure S2**.

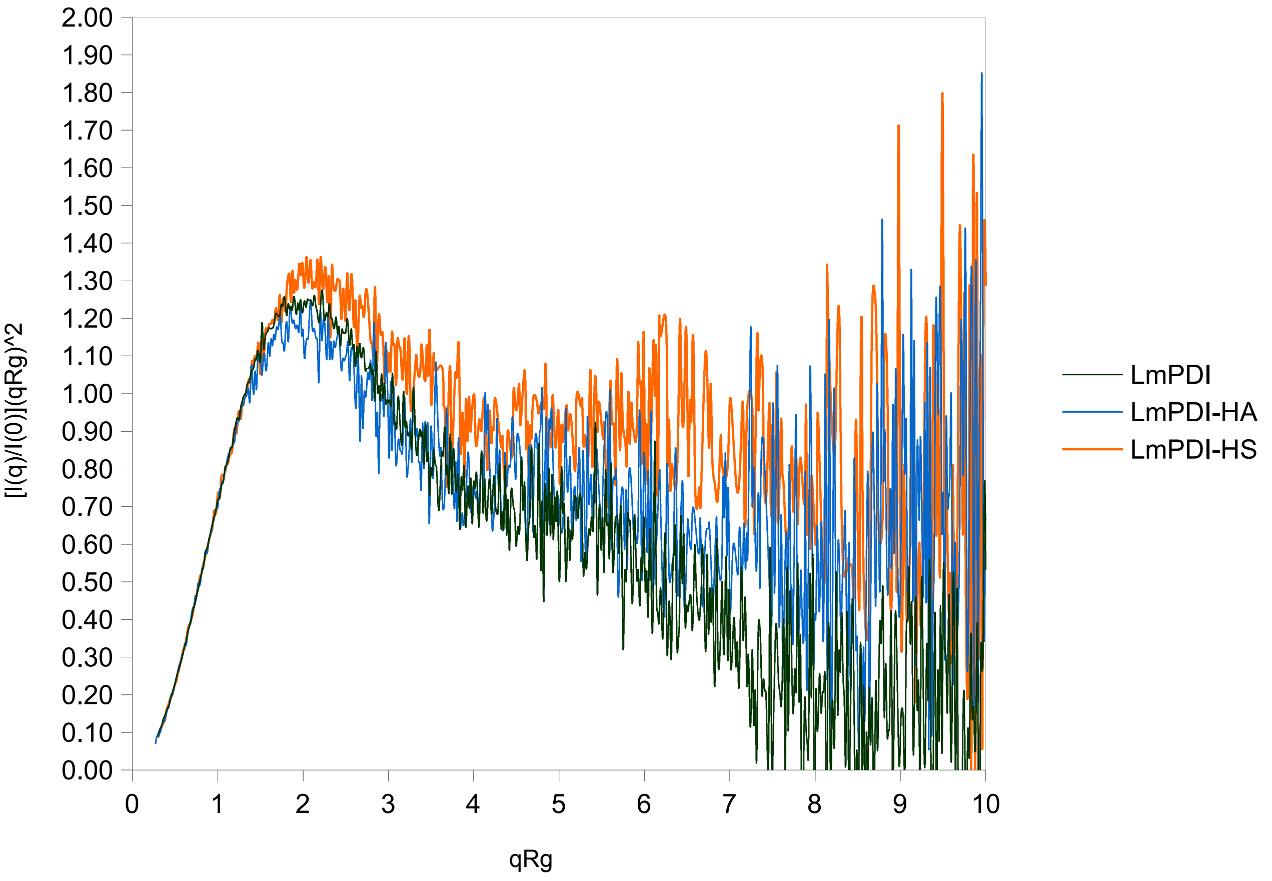

Supplementary Figure S5. Examples of reduction kinetics of di-eosin-GSSG monitored by the fluorescence emission signal of eosin at 520 nm. Error bars are the deviations from the average of 4 to 6 experiments per each concentration of protein.

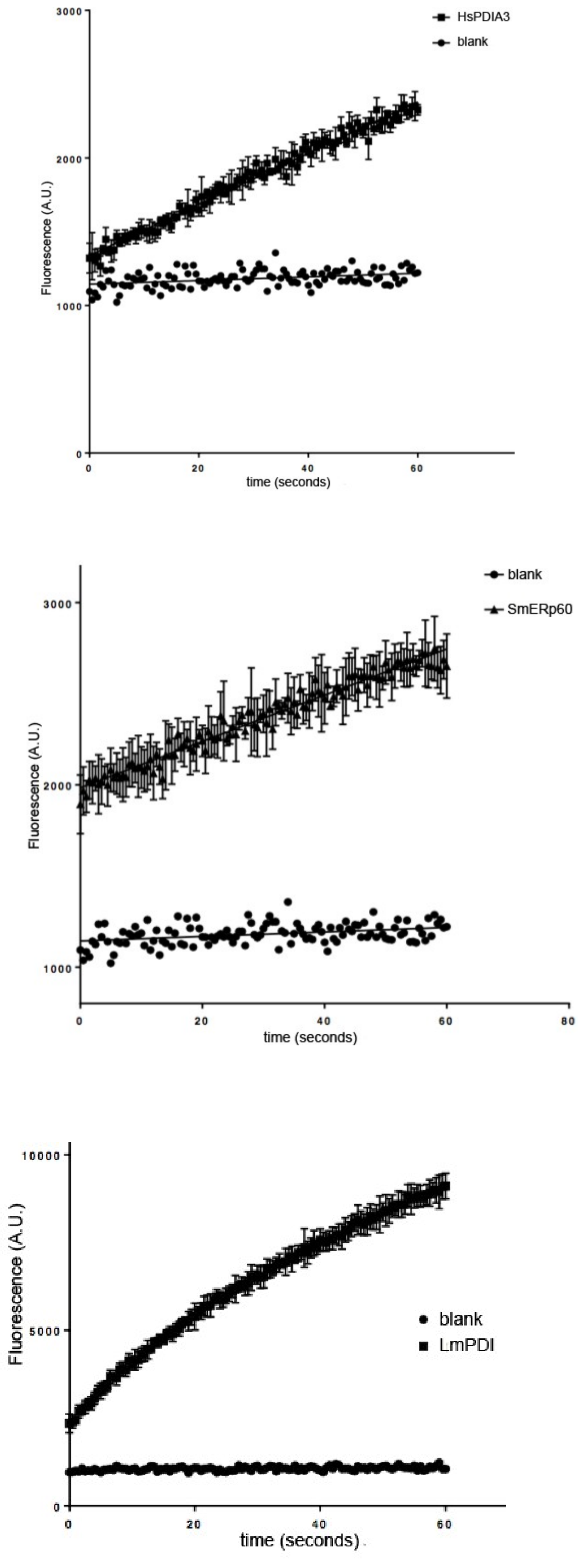

Supplementary Figure S6. Redox activity of LmPDI (top) and SmERp60 (bottom) measured at 37°C with the turbidimetric assay, by monitoring insulin precipitation at 650 nm with a spectrophotometer Cary 60 (Agilent). The protein concentration was 500 nM in 100 mM potassium phosphate buffer pH 7.0, EDTA 1 mM; insulin was 200 µM, DTT 5 µM. Increasing concentrations of bacitracin (SIGMA) were added to the mixture, from 0.5 to 2 mM.

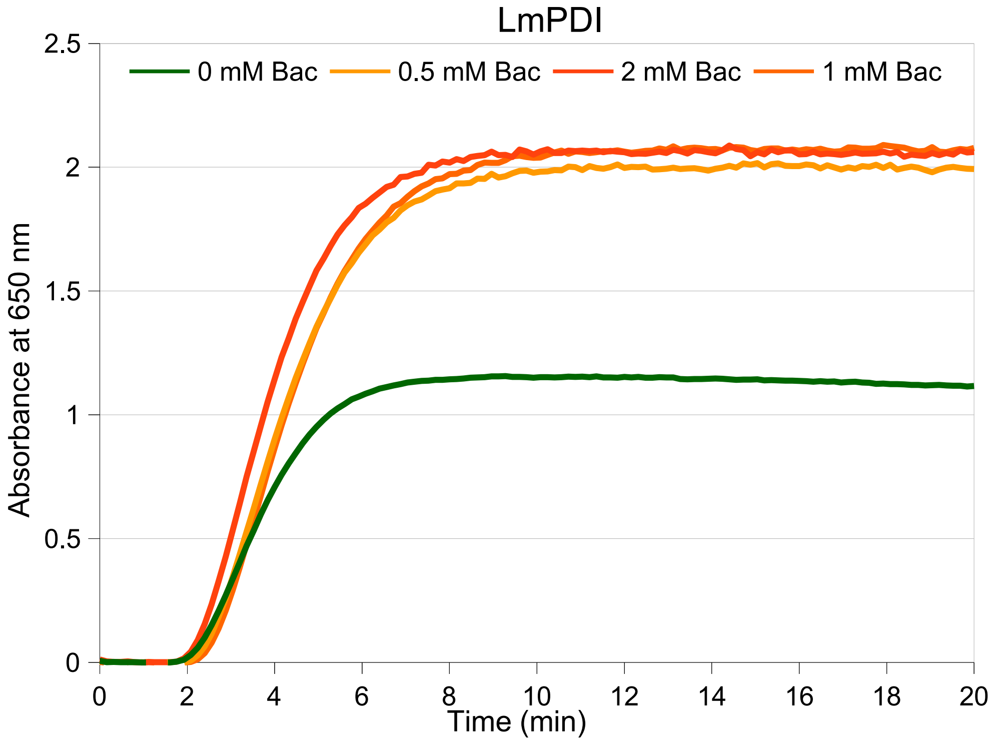

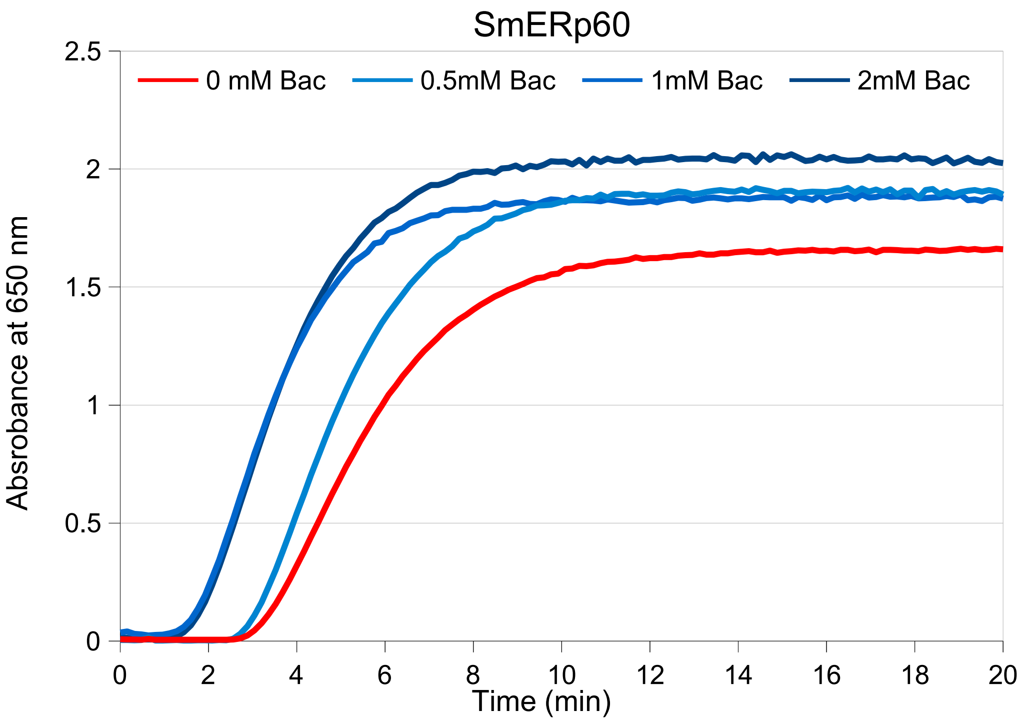

Supplementary Figure S7. Analysis by Coomassie stained SDS PAGEs of purified SmERp60 and LmPDI. Running gels were forecasted at 15% acrylamide; samples were denatured with 0.1% SDS and reduced with 2 mM β-mercaptoethanol. Running conditions: 100 V, in Tris-Gly-SDS running buffer, 20 °C.
Symbols legend: *, fusion proteins; →, cleaved proteins.

Panel A. Purified **SmERp60**. Lane 1: GST-SmERp60 bound to the GSH-Sepharose resin (GE-Healthcare) at the end of the purification; lane 2: elution of SmERp60 after overnight incubation with bovine thrombin; M: molecular weight markers (Thermo Fisher Page Ruler Plus, indicated masses are in kDa).
Panel B. Purified **LmPDI**. Lane 1: His-FLAG-LmPDI full length; lane 2: overnight incubation with enterokinase (Roche); lane 3: eluted LmPDI from the inverse affinity chromatography; M: molecular weight markers (Thermo Fisher Page Ruler Plus).

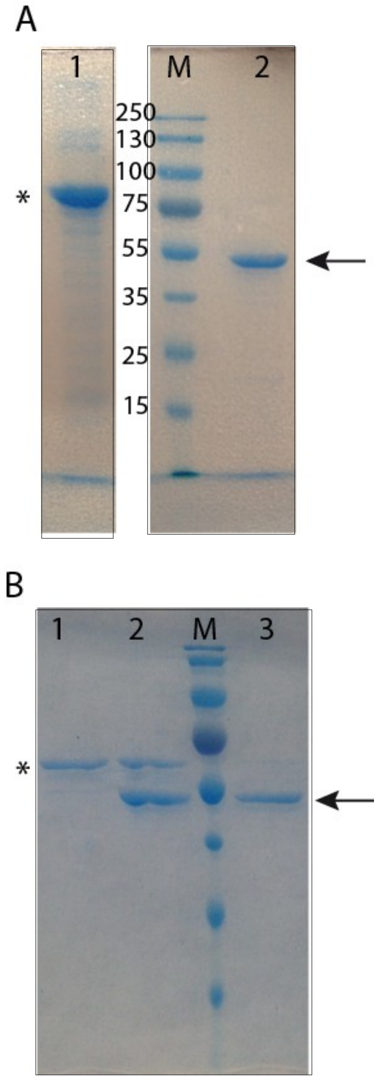

Original picture of the SDS-PAGE shown in Figure S7A. Lane 1: input GST-SmERp60; lane 2: 30' incubation with bovine thrombin; lane 3: 1h incubation; lane 4: 2h incubation; lane 5: 3h incubation; lane 6: 4h incubation; lane 7: overnight incubation; lane 8: Molecular weight markers; lane 9-10: digested SmERp60 eluted form the reverse affinity chromatography.

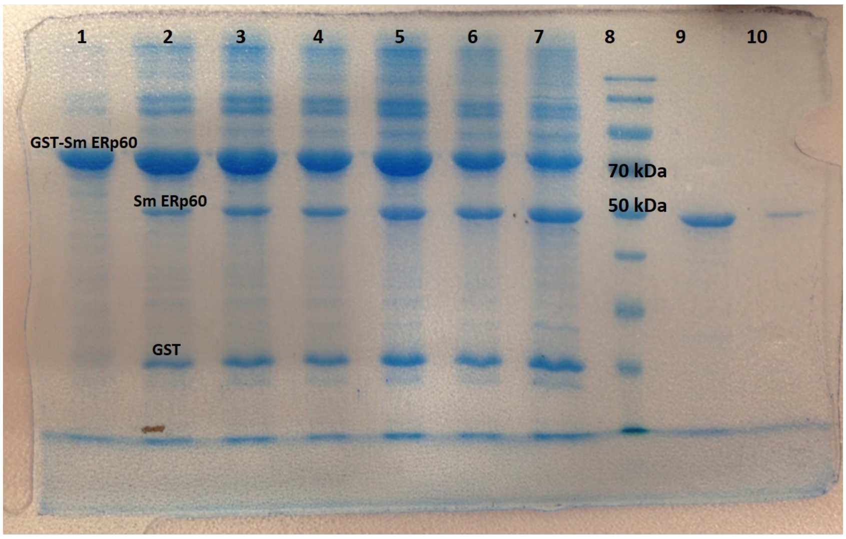

Original Picture of the SDS-Page shown in Figure S7B. Lane 1: HisFLAG-LmPDI; lane 2: lane 2: 1h incubation with enterokinase; lane 3: 2h incubation; lane 4: 4h incubation; lane 5: 8h incubation; lane 6: overnight incubation; lane 7: Molecular weight markers; lane 8: digested LmPDI eluted form the reverse affinity chromatography; lane 9: Ni-NTA beads after elution.

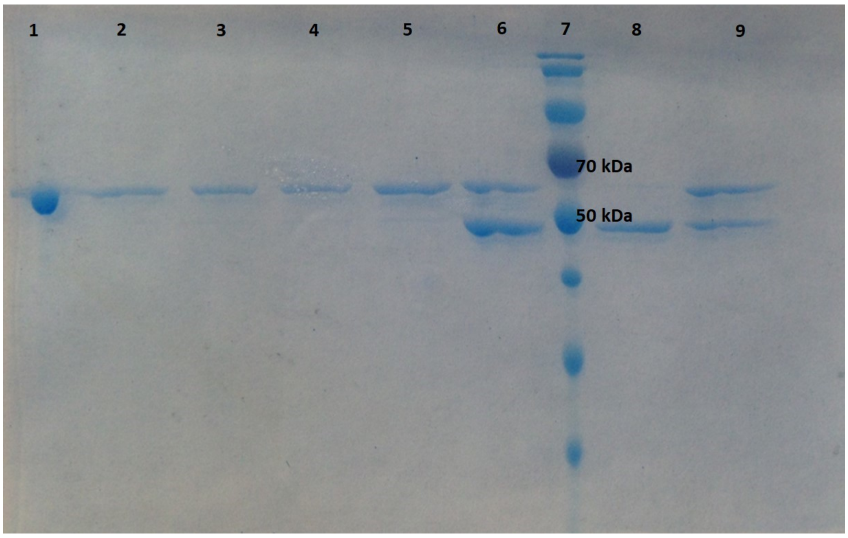

Supplement: Supplementary file 1 — Supplementary Information [file 41598_2019_45709_MOESM1_ESM.pdf]
